# Supplementary material for: COVID-19 severity and mortality in multiple sclerosis are not associated with immunotherapy: Insights from a nation-wide Austrian registry
Source: PLoS One. 2021 Jul 27;16(7):e0255316. doi: 10.1371/journal.pone.0255316 (PMC8315529; doi:10.1371/journal.pone.0255316)
Supplement: S1 Table — ATZ: alemtuzumab, CHD: coronary heart disease., CKD: Chronic kidney disease, CLA: cladribine, COPD: chronic obstructive pulmonary disease, DMF: dimethyl fumarate, DMT: disease modifying treatment, EDSS: Expanded disability status scale, FTY: fingolimod, F: female, GLA: glatiramer acetate, IFN: interferon beta, M: male, NTZ: natalizumab, OCR: ocrelizumab, PCR: SARS-CoV-2-polymerase-chain-reaction, RTX: rituximab, TERI: teriflunomide. (DOCX) [file pone.0255316.s001.docx]

**S1 Table. Characteristics of all 126 patients included.**

| **No** | **Age (years)** | **Sex** | **MS course** | **Disease duration (years)** | **Comorbidities** | **EDSS** | **DMT** | **COVID diagnosis** | **Severity** | **Management** | **Outcome at last follow-up** |
| --- | --- | --- | --- | --- | --- | --- | --- | --- | --- | --- | --- |
| 1 | 43 | M | SPMS | 21 | steatosis hepatis | 3,5 | None | Clin | Mild | Ambulatory No specific treatment | Recovered |
| 2 | 47 | M | RRMS | 14 | arterial hypertension | 1,0 | OCR | PCR | Mild | Ambulatory No specific treatment | Recovered |
| 3 | 29 | M | RRMS | 4 | None | 0 | DMF | PCR | Mild | Ambulatory No specific treatment | Recovered |
| 4 | 76 | M | SPMS | 30 | arterial hypertension CHD COPD | 7,5 | None | PCR | Mild | Ambulatory No specific treatment | Recovered |
| 5 | 54 | M | SPMS | 28 | None | 4,0 | None | PCR | Mild | Ambulatory No specific treatment | Recovered |
| 6 | 21 | F | RRMS | 1 | diabetes | 1,0 | OCR | PCR | Mild | Ambulatory No specific treatment | Recovered |
| 7 | 23 | M | RRMS | 8 | None | 1,0 | FTY | PCR | Mild | Ambulatory No specific treatment | Recovered |
| 8 | 32 | M | RRMS | 2 | obesity | 1,0 | NTZ | PCR | Critical | ICU mechanical ventilation  Antibiotics, corticosteroids  Rekonvalescent plasma | Recovered |
| 9 | 30 | F | RRMS | 5 | acute MS relapse | 2,0 | None | PCR | Asymptomatic | Ambulatory No specific treatment | Recovered |
| 10 | 48 | F | RRMS | 20 | None | 1,5 | FTY | PCR | Mild | Ambulatory No specific treatment | Recovered |
| 11 | 49 | F | RRMS | 20 | arterial hypertension depression | 4,0 | DMF | PCR | Mild | Hospitalized No specific treatment | Recovered |
| 12 | 25 | F | RRMS | 4 | None | 6,0 | RTX | PCR | Severe | Hospitalized Oxygen Antibiotics, corticosteroids | Recovered |
| 13 | 56 | F | SPMS | 19 | idiopathic pulmonal fibrosis deep venous thrombosis (oral anticoagulation) | 5,5 | None | PCR | Severe | Hospitalized Oxygen Corticosteroids | Recovered |
| 14 | 36 | M | RRMS | 1 | None | 2,5 | OCR | PCR | Mild | Ambulatory No specific treatment | Recovered |
| 15 | 26 | F | RRMS | 1 | depression | 1,0 | OCR | PCR | Mild | Ambulatory No specific treatment | Recovered |
| 16 | 34 | F | RRMS | 10 | pregnant asthma | 1,0 | None | PCR | Mild | Ambulatory No specific treatment | Recovered |
| 17 | 38 | M | RRMS | 15 | None | 2,0 | FTY | PCR | Mild | Ambulatory No specific treatment | Recovered |
| 18 | 42 | M | RRMS | 13 | obesity | 1,0 | FTY | PCR | Mild | Ambulatory No specific treatment | Recovered |
| 19 | 30 | F | RRMS | 2 | None | 0 | DMF | PCR | Mild | Ambulatory No specific treatment | Recovered |
| 20 | 53 | F | RRMS | 19 | None | 1,5 | NTZ | PCR | Mild | Ambulatory No specific treatment | Recovered |
| 21 | 39 | F | RRMS | 14 | pituitary adenoma | 1,0 | GLA | PCR | Mild | Ambulatory No specific treatment | Recovered |
| 22 | 50 | F | RRMS | 9 | None | 2,5 | NTZ | Clin+Ab | Mild | Ambulatory No specific treatment | Recovered |
| 23 | 35 | F | RRMS | 5 | None | 0 | DMF | Clin | Mild | Ambulatory No specific treatment | Recovered |
| 24 | 57 | M | RRMS | 12 | None | 4,0 | GLA | PCR | Mild | Ambulatory No specific treatment | Recovered |
| 25 | 32 | F | RRMS | 3 | None | 2,0 | RTX | PCR | Severe | Hospitalized Oxygen Rekonvalescent plasma | Recovered |
| 26 | 64 | F | SPMS | 38 | arterial hypertension depression epilepsy Mb. Osler | 6,0 | None | PCR | Critical | Hospitalized Oxygen Antibiotics, corticosteroids | Deceased |
| 27 | 56 | F | SPMS | 26 | None | 4,0 | TERI | PCR | Mild | Ambulatory No specific treatment | Recovered |
| 28 | 79 | M | SPMS | 28 | arterial hypertension CKD depression diabetes | 9,0 | None | PCR | Severe | Hospitalized Oxygen Antibiotics, corticosteroids | Improved |
| 29 | 59 | F | RRMS | 27 | None | 2,0 | None | PCR | Mild | Ambulatory No specific treatment | Recovered |
| 30 | 26 | F | RRMS | 2 | None | 1,0 | OCR | PCR | Mild | Ambulatory No specific treatment | Recovered |
| 31 | 42 | F | RRMS | 2 | depression | 3,5 | OCR | PCR | Mild | Ambulatory No specific treatment | Recovered |
| 32 | 55 | F | PPMS | 1 | None | 3,5 | OCR | PCR | Mild | Ambulatory No specific treatment | Recovered |
| 33 | 64 | F | PPMS | 2 | None | 4,0 | None | PCR | Mild | Ambulatory No specific treatment | Recovered |
| 34 | 21 | F | RRMS | 1 | tension headache | 0 | DMF | PCR | Mild | Ambulatory No specific treatment | Recovered |
| 35 | 32 | F | RRMS | 7 | Mb. Hashimoto | 0 | NTZ | PCR | Mild | Ambulatory No specific treatment | Recovered |
| 36 | 45 | F | RRMS | 6 | None | 1,0 | None | PCR | Mild | Ambulatory No specific treatment | Recovered |
| 37 | 32 | F | RRMS | 15 | St.p. aneurysmatic subarachnoidal hemorrhage | 0 | FTY | PCR | Mild | Ambulatory No specific treatment | Recovered |
| 38 | 49 | M | RRMS | 17 | None | 2,5 | None | PCR | Mild | Ambulatory No specific treatment | Recovered |
| 39 | 45 | F | RRMS | 1 | None | 2,5 | OCR | PCR | Mild | Ambulatory No specific treatment | Recovered |
| 40 | 59 | F | SPMS | 23 | arterial hypertension CKD pulmonary embolism (oral anticoagulation) | 7,0 | RTX | PCR | Critical | ICU mechanical ventilation  Antibiotics, corticosteroids | Deceased |
| 41 | 41 | M | RRMS | 18 | None | 6,0 | OCR | PCR | Mild | Ambulatory No specific treatment | Recovered |
| 42 | 42 | F | RRMS | 6 | None | 4,5 | GLA | PCR | Mild | Ambulatory No specific treatment | Recovered |
| 43 | 35 | F | RRMS | 8 | None | 0 | IFN | PCR | Mild | Ambulatory No specific treatment | Recovered |
| 44 | 41 | F | RRMS | 12 | None | 1,5 | TERI | PCR | Mild | Ambulatory No specific treatment | Recovered |
| 45 | 30 | M | RRMS | 16 | depression | 1,5 | None | PCR | Mild | Hospitalized Lopinavir/Ritonavir (Kaletra) | Recovered |
| 46 | 36 | F | RRMS | 5 | None | 1,5 | OCR | PCR | Mild | Ambulatory No specific treatment | Recovered |
| 47 | 56 | F | RRMS | 9 | arterial hypertension | 1,0 | IFN | PCR | Mild | Ambulatory No specific treatment | Recovered |
| 48 | 30 | M | RRMS | 6 | None | 0,0 | FTY | PCR | Mild | Ambulatory No specific treatment | Recovered |
| 49 | 31 | F | RRMS | 10 | None | 2,0 | FTY | PCR | Mild | Ambulatory No specific treatment | Recovered |
| 50 | 28 | M | RRMS | 6 | asthma CKD | 2,5 | DMF | PCR | Mild | Hospitalized Antibiotics, corticosteroids | Recovered |
| 51 | 32 | M | RRMS | 3 | None | 1,0 | DMF | PCR | Mild | Ambulatory No specific treatment | Recovered |
| 52 | 31 | M | RRMS | 5 | None | 1,0 | None | PCR | Asymptomatic | Ambulatory No specific treatment | Recovered |
| 53 | 40 | F | SPMS | 18 | None | 3,5 | RTX | PCR | Mild | Ambulatory No specific treatment | Recovered |
| 54 | 57 | M | PPMS | 2 | None | 3,0 | None | PCR | Mild | Ambulatory No specific treatment | Recovered |
| 55 | 28 | M | RRMS | 2 | None | 1,0 | DMF | PCR | Mild | Ambulatory No specific treatment | Recovered |
| 56 | 25 | F | RRMS | 5 | None | 1,0 | NTZ | PCR | Mild | Ambulatory No specific treatment | Recovered |
| 57 | 40 | F | RRMS | 4 | obesity | 4,5 | NTZ | PCR | Mild | Ambulatory No specific treatment | Recovered |
| 58 | 24 | F | RRMS | 4 | None | 0 | FTY | PCR | Mild | Ambulatory No specific treatment | Recovered |
| 59 | 50 | M | RRMS | 19 | obesity | 1,5 | IFN | PCR | Mild | Ambulatory No specific treatment | Recovered |
| 60 | 58 | F | RRMS | 13 | None | 2,0 | FTY | PCR | Mild | Ambulatory No specific treatment | Recovered |
| 61 | 32 | F | RRMS | 16 | systemic lupus erythematodes | 1,5 | None | PCR | Mild | Ambulatory No specific treatment | Recovered |
| 62 | 49 | M | RRMS | 16 | Obesity | 1,0 | IFN | PCR | Mild | Ambulatory No specific treatment | Recovered |
| 63 | 59 | F | PPMS | 12 | None | 2,0 | None | PCR | Mild | Ambulatory No specific treatment | Recovered |
| 64 | 38 | F | RRMS | 1 | None | 1,0 | FTY | PCR | Mild | Ambulatory No specific treatment | Recovered |
| 65 | 38 | F | RRMS | 11 | arterial hypertension | 4,0 | GLA | PCR | Mild | Ambulatory No specific treatment | Recovered |
| 66 | 41 | F | RRMS | 17 | None | 1,0 | DMF | PCR | Mild | Ambulatory No specific treatment | Recovered |
| 67 | 24 | F | RRMS | 3 | None | 1,0 | IFN | PCR | Mild | Ambulatory No specific treatment | Recovered |
| 68 | 36 | M | RRMS | 2 | None | 2,0 | FTY | PCR | Mild | Ambulatory No specific treatment | Recovered |
| 69 | 57 | F | PPMS | 3 | None | 6,0 | OCR | PCR | Mild | Ambulatory No specific treatment | Recovered |
| 70 | 21 | F | RRMS | 16 | None | 1,5 | NTZ | PCR | Mild | Ambulatory No specific treatment | Recovered |
| 71 | 50 | F | RRMS | 1 | None | 1,0 | None | PCR | Mild | Ambulatory No specific treatment | Recovered |
| 72 | 52 | F | RRMS | 2 | None | 0 | DMF | PCR | Mild | Ambulatory No specific treatment | Recovered |
| 73 | 48 | F | RRMS | 26 | None | 1,0 | None | PCR | Mild | Ambulatory No specific treatment | Recovered |
| 74 | 58 | M | SPMS | 35 | mitral insufficiency supraventricular Tachycardia | 6,5 | RTX | PCR | Severe | Hospitalized Oxygen Antibiotics, corticosteroids | Recovered |
| 75 | 57 | F | SPMS | 20 | None | 4,0 | None | PCR | Mild | Ambulatory No specific treatment | Recovered |
| 76 | 37 | F | RRMS | 3 | None | 1,5 | GLA | PCR | Mild | Ambulatory No specific treatment | Recovered |
| 77 | 46 | F | RRMS | 3 | None | 1,0 | None | PCR | Mild | Ambulatory No specific treatment | Recovered |
| 78 | 36 | F | RRMS | 5 | None | 0 | NTZ | PCR | Mild | Ambulatory No specific treatment | Recovered |
| 79 | 42 | F | PPMS | 8 | arterial hypertension obesity prothrombin mutation | 4,5 | OCR | Clin | Mild | Ambulatory No specific treatment | Recovered |
| 80 | 27 | F | RRMS | 5 | None | 0 | DMF | PCR | Mild | Ambulatory No specific treatment | Improved |
| 81 | 56 | F | RRMS | 24 | None | 3 | None | Clin | Mild | Ambulatory No specific treatment | Recovered |
| 82 | 27 | F | RRMS | 6 | None | 0 | DMF | PCR | Mild | Ambulatory No specific treatment | Recovered |
| 83 | 64 | M | RRMS | 17 | COPD | 2,0 | None | PCR | Mild | Ambulatory No specific treatment | Recovered |
| 84 | 48 | F | RRMS | 7 | None | 2,0 | None | PCR | Mild | Ambulatory No specific treatment | Recovered |
| 85 | 61 | M | PPMS | 26 | arterial hypertension CHD CKD COPD | 8,0 | None | PCR | Critical | Hospitalized Oxygen,  Antibiotics, corticosteroids | Deceased |
| 86 | 45 | F | RRMS | 2 | None | 1,0 | GLA | PCR | Mild | Ambulatory No specific treatment | Recovered |
| 87 | 49 | F | RRMS | 27 | None | 1,0 | FTY | Clin | Mild | Ambulatory No specific treatment | Recovered |
| 88 | 79 | M | PPMS | 22 | arterial hypertension CKD diabetes | 8,5 | None | PCR | Critical | Hospitalized Oxygen Patient decree | Deceased |
| 89 | 47 | F | RRMS | 4 | None | 0 | None | PCR | Mild | Ambulatory No specific treatment | Recovered |
| 90 | 33 | M | RRMS | 6 | None | 0 | DMF | PCR | Mild | Ambulatory No specific treatment | Recovered |
| 91 | 33 | M | RRMS | 1 | None | 1,0 | DMF | PCR | Mild | Ambulatory No specific treatment | Recovered |
| 92 | 31 | M | RRMS | 17 | None | 4,0 | DMF | PCR | Mild | Ambulatory No specific treatment | Recovered |
| 93 | 35 | M | RRMS | 5 | None | 0 | DMF | PCR | Mild | Ambulatory No specific treatment | Recovered |
| 94 | 39 | F | RRMS | 11 | None | 0 | IVIG | PCR | Mild | Ambulatory No specific treatment | Recovered |
| 95 | 30 | F | RRMS | 1 | None | 1,0 | RTX | PCR | Mild | Ambulatory No specific treatment | Recovered |
| 96 | 33 | F | RRMS | 11 | None | 1,0 | NTZ | PCR | Mild | Ambulatory No specific treatment | Recovered |
| 97 | 36 | F | RRMS | 8 | None | 2,0 | CLA | PCR | Mild | Ambulatory No specific treatment | Recovered |
| 98 | 63 | F | SPMS | 27 | None | 4,5 | GLA | PCR | Critical | ICU mechanical ventilation  Antibiotics, corticosteroids | Recovered |
| 99 | 42 | F | RRMS | 28 | None | 2,5 | CLA | PCR | Mild | Ambulatory No specific treatment | Recovered |
| 100 | 52 | M | RRMS | 9 | None | 1,5 | FTY | PCR | Mild | Ambulatory No specific treatment | Recovered |
| 101 | 45 | M | RRMS | 9 | None | 2,0 | GLA | PCR | Mild | Ambulatory No specific treatment | Recovered |
| 102 | 63 | F | RRMS | 15 | None | 1,0 | None | PCR | Mild | Ambulatory No specific treatment | Recovered |
| 103 | 35 | M | RRMS | 4 | dermatomyositis | 0 | None | PCR | Mild | Ambulatory No specific treatment | Recovered |
| 104 | 58 | F | RRMS | 5 | None | 1,5 | DMF | PCR | Mild | Ambulatory No specific treatment | Recovered |
| 105 | 23 | F | RRMS | 1 | None | 0 | GLA | PCR | Mild | Ambulatory No specific treatment | Recovered |
| 106 | 29 | F | RRMS | 2 | diabetes | 1,5 | ATZ | PCR | Mild | Hospitalized Oxygen,  Antibiotics, corticosteroids Remdesevir | Recovered |
| 107 | 59 | F | SPMS | 28 | None | 2,5 | GLA | PCR | Mild | Ambulatory No specific treatment | Recovered |
| 108 | 48 | F | RRMS | 16 | None | 4,0 | FTY | PCR | Mild | Ambulatory No specific treatment | Recovered |
| 109 | 65 | F | SPMS | 65 | Oosteoporosis | 6,5 | None | PCR | Mild | Ambulatory No specific treatment | Recovered |
| 110 | 27 | M | RRMS | 1 | hyperlipidemia | 0 | GLA | PCR | Mild | Ambulatory No specific treatment | Recovered |
| 111 | 61 | F | SPMS | 21 | None | 7,5 | None | PCR | Mild | Ambulatory No specific treatment | Recovered |
| 112 | 36 | F | RRMS | 14 | None | 2,0 | NTZ | PCR | Mild | Ambulatory No specific treatment | Recovered |
| 113 | 53 | F | SPMS | 19 | None | 3,0 | None | PCR | Mild | Ambulatory No specific treatment | Recovered |
| 114 | 53 | F | RRMS | 12 | None | 2,0 | None | PCR | Mild | Ambulatory No specific treatment | Recovered |
| 115 | 45 | F | RRMS | 22 | None | 2,5 | FTY | PCR | Mild | Ambulatory No specific treatment | Recovered |
| 116 | 26 | F | RRMS | 3 | obesity | 1,5 | DMF | PCR | Mild | Ambulatory No specific treatment | Recovered |
| 117 | 27 | M | RRMS | 12 | None | 2,0 | RTX | PCR | Asymptomatic | Ambulatory No specific treatment | Recovered |
| 118 | 54 | F | SPMS | 15 | arterial hypertension | 4,5 | IFN | PCR | Mild | Ambulatory No specific treatment | Recovered |
| 119 | 41 | F | RRMS | 19 | osteoporosis | 3,5 | RTX | PCR | Mild | Ambulatory No specific treatment | Recovered |
| 120 | 45 | F | RRMS | 16 | depression | 2,5 | DMF | PCR | Mild | Ambulatory No specific treatment | Recovered |
| 121 | 50 | F | SPMS | 33 | depression | 4,0 | None | PCR | Mild | Ambulatory No specific treatment | Recovered |
| 122 | 52 | F | SPMS | 15 | depression | 6,5 | RTX | PCR | Asymptomatic | Ambulatory No specific treatment | Recovered |
| 123 | 58 | F | SPMS | 20 | deep venous thrombosis,  idiopathic pulmonal fibrosis | 6,0 | None | PCR | Severe | Hospitalized Oxygen,  Antibiotics, corticosteroids | Improved |
| 124 | 70 | F | RRMS | 1 | arterial hypertension Rheumatoid arthritis | 2,0 | None | PCR | Mild | Ambulatory No specific treatment | Recovered |
| 125 | 29 | F | RRMS | 14 | autoimmune thyreoditis | 0 | ATZ | PCR | Mild | Ambulatory No specific treatment | Recovered |
| 126 | 53 | F | RRMS | 15 | depression | 0 | FTY | PCR | Mild | Ambulatory No specific treatment | Recovered |

ATZ: alemtuzumab

CHD: coronary heart disease.

CKD: Chronic kidney disease

CLA: cladribine

COPD: chronic obstructive pulmonary disease

DMF: dimethyl fumarate

DMT: disease modifying treatment

EDSS: Expanded disability status scale

FTY: fingolimod

F: female

GLA: glatiramer acetate

IFN: interferon beta

M: male

NTZ: natalizumab

OCR: ocrelizumab

PCR: SARS-CoV-2-polymerase-chain-reaction

RTX: rituximab

TERI: teriflunomide
