# Supplementary material for: COVID-19 severity and mortality in multiple sclerosis are not associated with immunotherapy: Insights from a nation-wide Austrian registry
Source: PLoS One. 2021 Jul 27;16(7):e0255316. doi: 10.1371/journal.pone.0255316 (PMC8315529; doi:10.1371/journal.pone.0255316)
Supplement: Attachment — Submitted filename: Rebutal_Letter_AutMuSC19_PlosOne.docx [file pone.0255316.s002.docx]

**Rebuttal to comments of reviewers and editor from initial submission (PONE-D-21-12442: COVID-19 severity and mortality in multiple sclerosis do not depend on immunotherapy: insights from a nation-wide Austrian registry)**

**Journal Requirements**

*1. When submitting your revision, we need you to address these additional requirements.Please ensure that your manuscript meets PLOS ONE's style requirements, including those for file naming.*

Response: Done.

*2. Please review your reference list to ensure that it is complete and correct. If you have cited papers that have been retracted, please include the rationale for doing so in the manuscript text, or remove these references and replace them with relevant current references. Any changes to the reference list should be mentioned in the rebuttal letter that accompanies your revised manuscript. If you need to cite a retracted article, indicate the article’s retracted status in the References list and also include a citation and full reference for the retraction notice.*

Response: Done.

*3. PLOS requires an ORCID iD for the corresponding author in Editorial Manager on papers submitted after December 6th, 2016. Please ensure that you have an ORCID iD and that it is validated in Editorial Manager. To do this, go to ‘Update my Information’ (in the upper left-hand corner of the main menu), and click on the Fetch/Validate link next to the ORCID field. This will take you to the ORCID site and allow you to create a new iD or authenticate a pre-existing iD in Editorial Manager.*

Response: Done.

*4. One of the noted authors is a group or consortium [AUT-MuSC investigators]. In addition to naming the author group, please list the individual authors and affiliations within this group in the acknowledgments section of your manuscript. Please also indicate clearly a lead author for this group along with a contact email address.*

Response: Done.

*5. Thank you for stating the following in the Competing Interests section: "I have read the journal's policy and the authors of this manuscript have the following competing interests. Please confirm that this does not alter your adherence to all PLOS ONE policies on sharing data and materials, by including the following statement: "This does not alter our adherence to PLOS ONE policies on sharing data and materials.” (as detailed online in our guide for authors* [*http://journals.plos.org/plosone/s/competing-interests*](about:blank)*).  If there are restrictions on sharing of data and/or materials, please state these. Please note that we cannot proceed with consideration of your article until this information has been declared. Please include your updated Competing Interests statement in your cover letter; we will change the online submission form on your behalf.*

Response: Done.

**Reviewers' comments**

**Reviewer: 1**

*Comment: Bsteh et al present a timely, population-based study regarding COVID-19 severity and mortality in multiple sclerosis patients in Austria. The authors included 129 multiple sclerosis patients with COVID-19, 86.5% had a mild course, 9.5% a severe course and 3.2% died. According to the authors, COVID-19 prevalence of the study cohort lies well within the general population. Neither exposure to any diseases-modifying treatment nor exposure to specific immunosuppressive DMT were significantly associated with COVID-19 severity. The mansucript is well-written and the results are of interest. However, the manuscript would improve when addressing a few minor issues.*

Response: Thank you for this positive assessment of our study.

*Comment: 1. Methods/results and abstract/conclusions are slightly imbalanced. Correlation does not imply causation, and both the manuscript title and the conclusion "treatment decisions should be focused on treating MS rather than the pandemic" are rather strong for a population-based study (with power issues) including 'only' 129 multiple sclerosis patients. Furthermore, the authors should clearly state that (asymptomatic) COVID-19 patients with multiple sclerosis may have been systematically missed due to the study design. Please adapt accordingly.*

Response: Thank you for this comment. We agree and have made an effort to improve the balance of the manuscript (see title, abstract and discussion section)

*Comment: 2. In the methods section, the authors wrote that the reference category is "multiple sclerosis patients without disease-modifying treatment exposure". In order to increase readability, please mention the reference category also in the abstract, results and discussion when appropriate.*

Response: Thank you for this comment. We have mentioned the reference category as requested (see abstract, results and discussion section)

*Comment: 3. The authors state that any disease-modifying treatment exposure was not associated with COVID-19 severity. However, a recently published study suggests that interferon antibodies are present in COVID-19 patients with a life-threatening course, especially in men and older patients (DOI: 10.1126/science.abd4585). Therefore, substituting interferon(-beta) may be beneficial regarding COVID-19 severitiy. If feasible, the authors could carefully comment on the 6 (4.8%) multiple sclerosis patients receiving interferon-beta having mild (and not life-threatening) COVID-19 (according to Suppl Table 1).*

Response: Thank you again for this comment. While this is certainly an interesting aspect, we do not think that our data stemming from 6 patients treated with interferon beta preparations allows for an evidence-based or sufficiently informed comment on the potential role of autoantibodies against IFN-alpha2 and IFN-ω.

*Comment: I thank the authors for their relevant scientific work.*

Response: We thank the reviewer for the diligent work and constructive criticism.

**Reviewer: 2**

*Comment: The authors report the findings on COVID-19 severity and mortality in MS patients investigated in a nation-wide Austrian registry. According to their results, outcome of COVID-19 is not modified by disease-modifying immunotherapy for MS. The methods are sound, the results are clearly presented, and the conclusions are justified.*

Response: Thank you for your positive assessment.

*Comment: 1) Methods. Please explain how diagnosis of COVID-19 was performed for the Austrian MS-COVID-19 registry.*

Response: Thank you for this comment. Diagnosis of was defined either by a positive SARS-CoV-2 polymerase chain reaction [PCR] or a clinical diagnosis supported by i) a subsequent positive SARS-CoV-2 antibody test or b) a positive SARS-CoV-2 PCR in a close contact person). This was clarified in the methods section (p4).

*Comment: 2) Methods. Please insert a table outlining the MS-COV-risk score recently developed by the authors. This would help a lot to better understand the findings.*

Response: Thank you for this suggestion. We have now included a table outlining the MS-COV-risk score as requested.

*Comment: 3) Table 1. please also show range for age*

Response: We have added range for age as requested.

*Comment: 4) Page 6, punctuation after reference [12]*

Response: Thank you for spotting this error. We have corrected the punctuation accordingly.
